# High‐Salt Diet Links Gut Microbiota, Intestinal Barrier Function, and Macrophage Responses

**DOI:** 10.1002/eji.70234

**Published:** 2026-07-22

**Authors:** Akram Abdulkadyrov, Marie‐Louise Diefenbach‐Wilke, Elvira Mass, Maria Francesca Viola

**Affiliations:** ^1^ Developmental Biology of the Immune System, Life and Medical Sciences (LIMES) Institute University of Bonn Bonn Germany

## Abstract

The Global North is increasingly exposed to a Western diet characterized by high fat, sugar, and salt content. Excess dietary salt has been linked to cardiovascular disease and hypertension and can accumulate in multiple tissues, exerting local immunomodulatory effects. Beyond these systemic consequences, a high‐salt diet (HSD) is associated with gut dysbiosis, which alters the production of microbial metabolites, such as short‐chain fatty acids (SCFAs), and compromises intestinal barrier integrity, thereby facilitating bacterial translocation and contributing to liver and kidney injury. These alterations are associated with inflammatory responses, although their direction and magnitude depend on dietary duration, microbial baseline composition, and experimental models. While most studies have focused on HSD‐induced modulation of T cell responses, emerging data highlight macrophages as underexplored mediators of HSD‐driven immune and metabolic effects. In this review, we summarize current knowledge on HSD‐induced alterations of the intestinal microbiota, microbial metabolites, gut barrier function and macrophage function, and discuss their potential interplay along the gut–liver axis. In addition, we highlight key gaps and challenges that must be addressed to improve translational relevance.

## Graphical abstract

This review examines how a high‐salt diet disrupts gut microbiota composition, impairs intestinal barrier integrity, and modulates macrophage function, with a focus on their interconnection along the gut‐liver axis. It highlights key methodological challenges, summarizes current mechanistic understanding, and identifies critical gaps for future research.

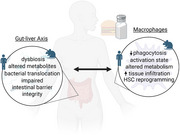

## 1 Introduction

Dietary patterns have shifted enormously in the last century, with a large part of the Global North now following what is known as a ‘Western diet’. The Western diet is characterized by a high content of saturated fats and sugars, combined with low fiber content, which has been shown to promote cardiovascular diseases, obesity, type 2 diabetes, and the loss of specific bacterial species that promote healthy gut physiology [1, 2]. An additional characteristic of the Western diet is a high salt content. Indeed, the U.S. Food and Drug Administration has estimated that the average daily sodium consumption in the US is approximately 3.4 g/day, while the recommended amounts of daily sodium intake by the World Health Organization (WHO) and the Dietary Guidelines for Americans are 2 and 2.3 g, respectively [3, 4]. In line with this, the WHO reported that in 2019, the mean population dietary sodium intake in the European and American regions was at least 70% higher than the recommended amount [5].

**FIGURE 1:**
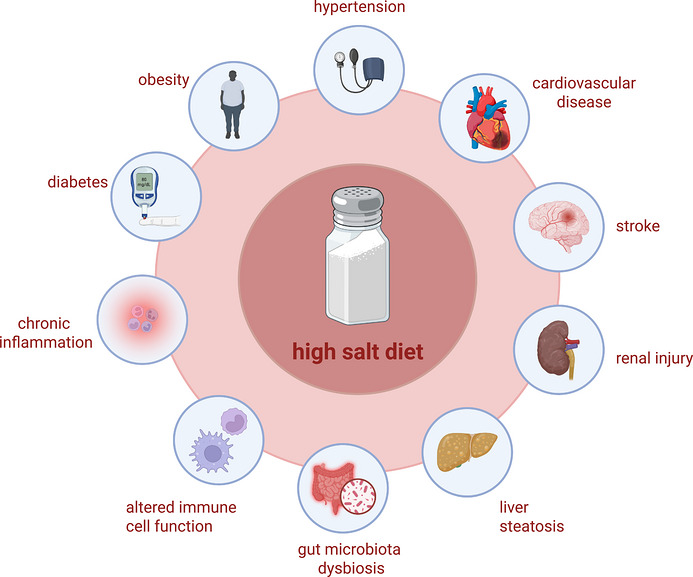
High‐salt diet (HSD) has been linked to noncommunicable diseases, changes in the intestinal microbiota, and altered immune function.

Over decades, research into the consequences of a Western diet has mainly focused on the high content of refined sugar and saturated fats, while the potential risks associated with high salt intake have largely been overlooked. However, growing evidence has linked high salt consumption to the rise in noncommunicable disorders such as obesity, hypertension, renal injuries, liver steatosis, and cardiovascular diseases (summarized in Figure 1) [6, 7, 8]. In mice, sodium accumulates in the skin, thymus, liver, spleen, and potentially the endothelial surface layers, while high‐salt diets have been shown to reduce salt content in the kidneys and bone marrow [7, 9, 10, 11]. These shifts in tissue salt storage may locally influence the function of surrounding cells. In addition, high salt intake can alter the composition of the gut microbiota, which in turn can indirectly lead to intestinal wall thinning and persistent low‐grade inflammation. Finally, higher salt concentrations can alter immune cell function, leading to aberrant immune activation in homeostasis and disease, and inducing long‐lasting effects in immune progenitors [6, 8, 12, 13].

This review summarizes the current state of knowledge on how a high‐salt diet (HSD) shapes the gut microbiome, intestinal barrier functions, and immune landscape, with a particular focus on macrophages and their functional interplay with microbial cues, to identify key knowledge gaps and outline future research directions.

## 2 Methodological Heterogeneity in HSD Interventions

Research into the effects of HSD has gained considerable momentum, though heterogeneity in experimental design warrants careful interpretation of findings across studies (Table 1). Key variables, including salt concentration, delivery route, exposure duration, and control conditions, differ substantially between studies, and understanding these differences is important for contextualizing results appropriately.

**TABLE 1:** Methodological approaches of high‐salt exposure in different model systems.

Study design	NaCl content in high‐salt condition	Model systems	NaCl content in control condition	Duration	References
In vivo	4% diet + 1% water	Mice:	0.3%–0.5% diet	2–8 weeks	[8, 9, 14, 15, 16, 17, 18, 19, 20, 21]
		C57BL/6J, FVB, FVB/N, Swiss/129Sv, C57BL/6Jola.			
		Rats:			
		Dahl/Salt‐sensitive rats			
	8% diet + 1% water	Mice:	0.4%–0.49% diet	2–8 weeks	[9, 13, 22]
		C57BL/6J			
		Rats;			
		Sprague–Dawley			
	8% diet	Mice:	0.3%–0.6% diet	6–22 weeks	[11, 23, 24, 25, 26, 27]
		C57BL/6J			
		Rats:			
		Sprague–Dawley, Wistar			
	8% diet + 0.9% water	Sprague–Dawley rats	<0.1% diet	2 weeks	[10]
	2% water	C57BL/6J mice	Tap water	8 weeks	[28]
	0.28% diet + 0.3% water	Sprague–Dawley rats	0.28% diet	8–12 weeks	[29]
	10% diet	Mongolian gerbils	0.32% diet	38 weeks	[30]
	9–15 g/day	Humans	5–6 g/day	2 weeks–50 days	[15, 31, 32]
In vitro	+20–70 mM NaCl (above control)	Mouse:	109.5–150 mM	3–96 h	[10, 13, 14, 15, 18, 20, 26, 31]
		BMDMs, peritoneal macrophages, splenic T cells			
		J774A.1 cells, RAW264.7 cells, MIN6 cells			
		Human:			
		primary MDMs, primary Th17 cells, primary monocytes			
	200 mM NaCl	Jurkat T cells, MCF‐7 cells	100 mM	6–48 h	[26, 33]
	300–1000 mM NaCl	RGM‐1 cells	150 mM	6 h	[34]

Standard rodent chow contains 0.3%–0.5% NaCl, and experimental HSD models typically employ 4%–10% NaCl—representing at least an eightfold increase above controls—whereas human excess salt intake reflects only an approximately twofold increase over recommended levels (Table 1) [5]. This concentration gap is a deliberate experimental strategy to isolate sodium‐driven signaling, but whether findings translate to the more modest sodium excess of human diets remains debated; notably, some human intervention studies have failed to replicate the pro‐inflammatory phenotypes observed in murine models, possibly reflecting interspecies differences in electrolyte metabolism and immune cell activation [35].

Delivery route adds further complication: some studies supplement solid chow with salt, others use salt‐enriched drinking water, and others combine both approaches, each producing distinct kinetics of sodium exposure and tissue distribution (summarized in Table 1). Exposure duration introduces additional complexity, as short‐term HSD primarily induces pro‐inflammatory responses, while long‐term HSD likely engages compensatory mechanisms that dampen initial inflammation [14, 15].

For in vitro studies, the field has largely converged on supplementing culture medium with an additional 40 mM NaCl as a working standard (summarized in Table 1). However, the frequent omission of osmolality controls represents an important limitation: since salt supplementation simultaneously increases extracellular sodium concentration and osmolality, attributing observed effects specifically to sodium requires parallel iso‐osmotic controls, typically mannitol or urea, that are not consistently included. Future research should prioritize the standardization of HSD intervention and include physiologically relevant exposure conditions.

## 3 HSD and the Intestinal Microbiota

The gastrointestinal tract is colonized by a highly complex microbial ecosystem, collectively referred to as the gut microbiota, which exerts profound effects on host physiology. In humans, microbial cells are present in approximately equal numbers to host cells, with the gastrointestinal tract estimated to harbor approximately 3.8 × 10^13^ bacterial cells, and the colon representing the site of the highest microbial density [36]. Beyond its remarkable abundance, the gut microbiome encompasses a genetic repertoire that vastly exceeds that of the human genome, providing extensive functional and metabolic potential. Importantly, dietary salt has emerged as a potent modulator of this ecosystem, with high sodium concentrations exerting differential bacteriostatic and growth‐promoting effects across microbial taxa, driving compositional shifts that compromise the functional integrity of the gut microbiota and its capacity to maintain host homeostasis [15, 17].

### 3.1 Effects of HSD on the Gut Microbiota

Numerous studies have described that HSD alters the gut microbiota composition and can induce gut dysbiosis by depleting beneficial bacteria [16, 17, 27, 28, 37, 38, 39]. Interestingly, HSD‐induced enteric dysbiosis does not appear to occur uniformly throughout the gut; instead, these alterations are region‐specific, as shown in a recent study by Hu et al. [28], demonstrating that HSD‐induced microbial shifts in the ileum, caecum, and colon differ from one another and vary in their intensity. These region‐specific changes in microbial composition are important to consider when interpreting study outcomes, as sampling sites may vary.

Reproducibility remains a significant challenge in microbiota research. SPF mouse models exclude defined pathogens but harbor a poorly defined, drift‐prone microbial community, and housing variables introduce additional compositional variation [40, 41]. Given the context‐dependency of HSD‐induced microbiota alterations and their susceptibility to experimental conditions, the following paragraphs focus on the microbial features reported most consistently across independent studies.

HSD‐induced gut dysbiosis operates across taxonomic levels. At the genus level, the most reproducible finding is the depletion of Lactobacillus spp., which occurs within days of increased salt intake, alongside reductions in *Bifidobacterium*, *Faecalibaculum*, and *Blautia*; genera collectively associated with immune‐competent short‐chain fatty acid (SCFA) production [15, 16, 17, 42]. HSD is consistently associated with *Prevotella* enrichment, an effect particularly robust under chronic exposure, whereas the relationship with *Bacteroides* is more variable across studies and contexts [16, 43]. The functional consequence of this shift is uncertain, given that elevated *Prevotella* has been associated with inflammatory conditions, yet certain species contribute to beneficial SCFA production [44, 45]. At the phylum level, findings are less consistent: while some studies report decreased *Bacillota* and increased *Bacteroidota*, others observe the opposite, alongside enrichment of *Pseudomonadota* and *Cyanobacteriota*, and the *Bacillota*/*Bacteroidota* ratio, frequently employed as a biomarker of microbial health, shows no consistent directional shift across disease contexts [16, 17, 27, 46]. Taken together, while HSD reproducibly perturbs microbial diversity and depletes key SCFA‐producing genera, a unified phylum‐level signature has not been established, and the overall outcome of dysbiosis remains dependent on the duration and intensity of salt exposure and the pre‐existing microbial landscape.

### 3.2 HSD‐Driven Modulation of Microbial Metabolites

Intestinal commensals are responsible for producing a wide range of metabolites that can regulate the host physiology and immune system. Human enzymes cannot digest numerous dietary fibers; thus, they remain inaccessible to host metabolism. However, they can serve as a substrate for gut bacteria, enabling the production of SCFAs such as propionate, acetate, and butyrate, mainly by carbohydrate‐fermenting bacteria in the distal colon [47, 48]. SCFAs play numerous physiological roles, including the regulation of gut motility and the modulation of intestinal barrier function, and can directly modulate immune cells [49, 50, 51, 52, 53]. Finally, SCFAs play a role in lipid and glucose metabolism, and low levels of SCFAs are associated with impaired insulin sensitivity and increased systemic inflammation [54].

High dietary salt disrupts SCFA production by negatively regulating SCFA‐producing bacteria, including *Bacteroides* and potentially *Ruminococcus*, supported by observations in mice, rats, and humans, and reducing sodium intake has been shown to increase circulating SCFAs [19, 24, 26, 43, 55, 56, 57]. Conversely, elevated acetate, propionate, and isobutyrate have been reported in HSD‐fed rats, and discrepancies across studies likely reflect differences in exposure duration, diet composition, and facility‐specific microbiota profiles. However, sex‐specific differences may also play a role, as decreasing dietary sodium to 2 g in hypertensive female participants with previously unregulated salt intake increased circulating SCFAs, whereas no significant changes in these SCFAs were observed in male participants [57]. A growing body of evidence indicates that sex differences actively shape the composition of the gut microbiome and are likely mediated via hormonal factors [58, 59, 60].

Parallel to SCFA production, the microbiota remodels the host bile acid (BA) pool, generating secondary metabolites that function as systemic immune and metabolic regulators. Commensal‐derived bile salt hydrolase (BSH) catalyzes the deconjugation of primary BAs, followed by further processing into secondary BAs in the colon, which function as endogenous ligands for a broad family of membrane and nuclear bile acid‐regulated receptors expressed throughout the body, including in innate immune cells [61, 62]. In addition to playing a crucial role in regulating hepatic lipid and glucose metabolism, secondary BAs can modulate the immune system and contribute to intestinal barrier integrity [63, 64, 65, 66].

Direct evidence for HSD‐driven changes in commensal bile acid metabolism remains scarce; however, the consistent reduction of *Lactobacillus*, a major BSH‐producing genus, across HSD studies suggests this warrants further investigation (Table 2) [67, 68, 69]. Of note, a study reported fecal secondary bile acid imbalance alongside disrupted intestinal tight junction expression and elevated systemic LPS, although the perinatal design introduces confounders related to lactation that limit direct extrapolation [70]. Another study found that HSD led to an increase in conjugated bile acids, further supporting HSD‐induced decreased BSH activity [69]. It should be noted that translational inference from murine HSD studies is constrained by fundamental interspecies differences in bile acid pool composition, which limit direct extrapolation to human physiology [62, 71].

**TABLE 2:** Overview of HSD‐mediated effects on gut microbiota and their metabolites.

**Modulated aspect**	**Direction under HSD**	**Previously linked health state(s)**	**Functional/Pathophysiological consequences**	**Reference(s)**
*Bacillota*	↑ or ↓ (inconsistent)	Obesity, metabolic syndrome, inflammation	Altered community structure; unreliable health biomarker	[16, 23, 27, 28, 72]
*Bacteroidota*	↑ or ↓ (inconsistent)	IBD, metabolic disease	Disturbed fiber metabolism; altered SCFA output	[16, 17, 23, 28, 56, 72]
*Bacillota*/*Bacteroidota* ratio	↑ or ↓ (study‐dependent)	Obesity, diabetes, cardiovascular disease	Poor predictor of gut health under HSD	[16, 17, 27, 28, 73, 74]
*Lactobacillus*	↓ (robust across studies, early onset)	Immune regulation, hypertension protection	Reduced SCFA production; altered BA metabolism; increased inflammation	[15, 17, 19, 71, 72]
*Barnesiella*	↑ (long‐term HSD)	Hepatic lipid accumulation	Potential compensatory expansion	[23]
*Christensenella*	↑ (long‐term HSD)	Obesity; obesity‐related metabolic disorders	Potential compensatory expansion	[21, 23]
*Bacteroides*	↑ or ↓ (study ‐dependent)	Metabolic disease, SCFA production	Shift in fiber fermentation	[16, 26, 43, 55, 56]
*Prevotella*	↑ (long‐term HSD)	IBD, mucosal inflammation	Pro‐inflammatory potential; SCFA production (species‐specific)	[16, 43, 44, 45]
*Bifidobacterium*	↑ or ↓ (study ‐dependent)	Gut barrier integrity, immune tolerance	Reduced acetate production	[17, 26, 27]
*Faecalibaculum*	↑ or ↓ (model ‐dependent)	Anti‐inflammatory effects	Lower butyrate availability	[17, 55]
*Blautia*	↓	Metabolic health, SCFA production	Reduced immune‐regulatory metabolites	[17]
*Ruminococcus*	↑ or ↓ (species‐dependent)	SCFA production, fiber degradation	Impaired fermentation capacity	[19, 26, 72]

Beyond SCFAs and bile acids, the gut microbiota produces a range of additional metabolites with established immunomodulatory and barrier‐regulatory functions, including tryptophan‐derived indole catabolites, which can modulate epithelial integrity and innate immune tone via the aryl hydrocarbon receptor, and trimethylamine N‐oxide (TMAO), a co‐metabolite linked to cardiovascular and inflammatory pathology [75, 76, 77]. Whether HSD‐driven dysbiosis alters the production of these metabolites and contributes to gut–liver axis dysfunction remains unexplored.

In summary, HSD reshapes the gut microbiota and its metabolic output, the key features of which are summarized in Table 2. How these changes impact host physiology, in particular intestinal barrier function and the immune system, is discussed in the following sections.

## 4 The Effect of HSD on Intestinal Barrier Integrity and Bacterial Translocation

The gastrointestinal tract carries out the delicate role of favoring nutrient absorption, while simultaneously acting as a barrier to bacteria and pathogens that are present within the intestinal lumen. In homeostasis, a layer of mucus protects the epithelial layer, and antimicrobial peptides (AMPs) are secreted into the mucus layer, thereby enhancing barrier function [78]. Finally, expression of tight junction proteins in the epithelial layer regulates the passage of molecules and pathogens through the epithelial layer into the tissue [72]. Disruption of these protective mechanisms can lead to bacterial translocation, defined as the passage of viable bacterial cells through the intestinal barrier to other sites, where they may exert immunomodulatory effects and potentially contribute to sepsis [73].

Numerous studies have suggested that HSD may lead to compromised gut barrier integrity and subsequent bacterial translocation, particularly to the kidneys [25, 28]. Nevertheless, in the context of HSD, distinguishing the relative contributions of microbiota, metabolites, and high salt intake to intestinal barrier dysfunction remains challenging. Beyond microbiota‐ and metabolite‐driven mechanisms, high salt concentrations cause osmotic stress with direct consequences for epithelial cell biology. Specifically, MAPK signaling pathways have been implicated as key regulators in response to osmotic stress, including p38, which contributes to activation of NFAT5 [74, 79, 80]. NFAT5 is a transcription factor directly activated by osmotic stress, driving the expression of osmoprotective genes such as aldose reductase (AR) and the betaine transporter (BGT1), which allows intracellular accumulation of osmolytes to preserve protein function, while also driving cytokine responses [81, 82, 83]. In addition, p38/MAPK signaling under osmotic stress orchestrates cytoskeletal remodeling, changes in cell volume, and cell proliferation [84, 85]. Collectively, these osmotic stress responses have the potential to compromise epithelial architecture and gene expression.

Consistent with these mechanisms, multiple studies in rodent models have observed HSD‐driven changes in mucosal barrier components. HSD reduces the expression of goblet cell–derived mucopolysaccharides in the intestine and decreases epithelial cell viability, in parallel with increased reactive oxygen species production in gastric mucosal cells [25, 34]. In addition, long‐term HSD intake reduces AMPs in the gut and is associated with decreased Mucin‐2 expression [25]. Moreover, HSD significantly decreases the expression of occludin, a key component of epithelial tight junctions, in the colon, and of Zonula occludens‐1 in both colon and ileum [25, 28]. Conversely, expression of Claudin‐2, which has been shown to drive increases in intestinal permeability, was increased in both the ileum and the colon [28, 86]. In a rat model of chronic kidney disease in combination with HSD, decreased expression of claudin‐4 and occludin, in addition to increased junction, was observed [29].

Elevated circulating d‐lactate and higher fecal albumin following HSD further support compromised barrier integrity, and in one study, the associated increase in intestinal permeability resulted in bacterial translocation to the kidney, with consequent apoptosis and impaired renal function [25, 28]. Collectively, these findings indicate that HSD can compromise intestinal barrier integrity and promote bacterial translocation, although the relevance for disease development remains to be fully clarified.

## 5 HSD and Macrophages

HSD affects immune function through multiple overlapping mechanisms, including shifts in microbial composition, metabolite production, bacterial translocation, and direct sodium sensing, making causal dissection in vivo difficult. Several in vitro studies nonetheless support direct sodium‐driven immune alterations, particularly in macrophages and T cells. In this section, we focus on the effects of HSD on macrophages, as the impact of high sodium on other immune cell types has been comprehensively reviewed elsewhere [87, 88].

As a first indication that high salt directly modulates macrophage function, an in vitro study demonstrated that, following high salt exposure, macrophages stimulated with IL‐4 and IL‐13 were no longer able to suppress effector T cell proliferation [20]. Macrophages also play a central role in the clearance of pathogens and cellular debris through receptors, including Fc gamma receptors (Fc𝛾Rs), the efferocytic receptors Tim4 and MerTK, as well as through pinocytosis and other nonreceptor‐mediated uptake mechanisms [89, 90, 91]. These functions are critical, as impaired phagocytosis can result in prolonged inflammation and may contribute to autoimmune disorders [92]. A recent study in bone marrow‐derived macrophages (BMDM) demonstrated that high salt exposure increased the expression of genes involved in chemotaxis, regulation of inflammatory responses, and macrophage migration, whereas genes associated with wound healing, chemokine production, IL‐1 production, and phagocytosis were downregulated [14]. Consistent with this, high salt reduced the expression of the inhibitory Fc𝛾R CD32b and of MerTK, while Tim4 expression was slightly increased [14]. Functionally, BMDMs and peritoneal macrophages showed a dose‐dependent reduction in phagocytic capacity in the presence of excess salt, indicating that high salt directly perturbs macrophage effector functions [14]. Notably, as CD32b plays an important role in limiting inflammatory responses, these findings further support the notion that high salt exposure may promote inflammation [93].

High salt has also been shown to affect mitochondrial respiration in macrophages, as basal and maximal oxygen consumption rates and cellular ATP levels were reduced in the presence of excess salt, together with an increase in aerobic glycolysis [13, 20, 31, 94]. Mechanistically, elevated extracellular sodium is taken up intracellularly and enters the mitochondrial matrix via the Na^+^/Ca^2^^+^ exchanger NCLX, where it interacts with inner mitochondrial membrane phospholipids to reduce membrane fluidity [31, 95, 96]. This leads to inhibition of complex II activity and a consequent reduction in mitochondrial membrane potential, oxygen consumption, and ATP production [31, 96, 97]. In macrophages specifically, this metabolic perturbation occurs independently of polarization state and is mediated, at least in part, by NCLX‐dependent sodium sensing at the inner mitochondrial membrane [31, 94, 98]. Given the tight coupling between mitochondrial oxidative phosphorylation and macrophage polarization state, sodium‐driven impairment of the electron transport chain may have direct consequences for macrophage inflammatory tone, shifting the balance away from reparative OXPHOS‐dependent programs toward pro‐inflammatory activation [99]. In addition, salt can act as a chemotactic stimulus for macrophages in vitro, a finding that is particularly intriguing in light of the heterogeneous distribution of sodium across tissues [7, 14].

**FIGURE 2:**
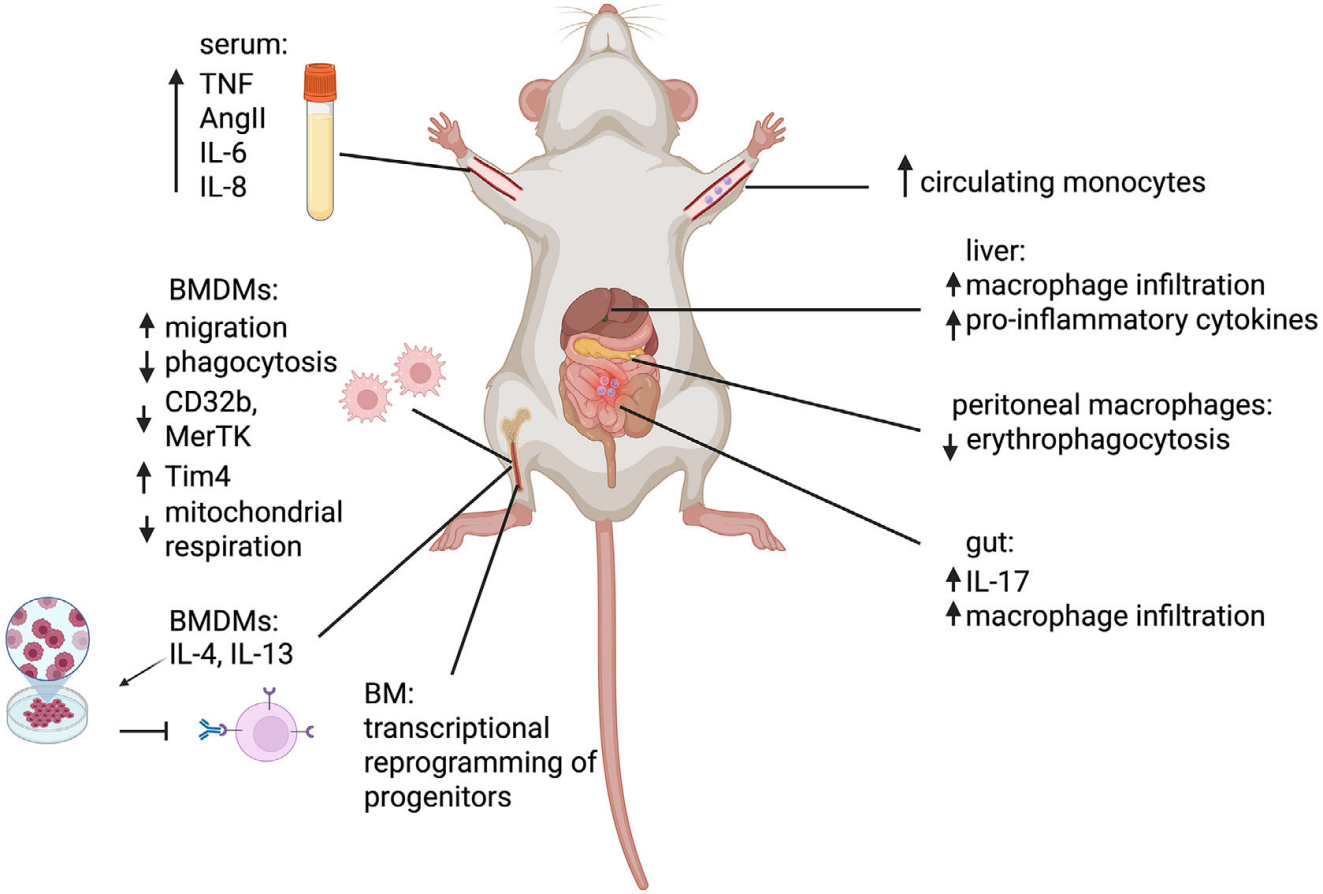
HSD induces reprogramming of progenitor cells in the bone marrow (BM), altered metabolism, and reduced phagocytosis in bone marrow‐derived macrophages (BMDMs).

Several in vivo studies in both humans and mice support key aspects of the in vitro findings described above. In mice, HSD impaired wound healing and reduced the expression of the anti‐inflammatory macrophage marker Fizz1, consistent with altered macrophage polarization in vivo [20]. Moreover, studies suggest a moderate increase in systemic and tissue inflammation under HSD conditions. In a small cohort of healthy male volunteers, periods of high salt intake were associated with a significantly increased number of circulating monocytes, whereas other leukocyte subsets remained unchanged [32]. In parallel, plasma concentrations of interleukin‐6 and interleukin‐17 were reduced during phases of low salt intake, while interleukin‐10 was increased [32]. Consistent with these observations, rodent studies have demonstrated that HSD increased serum levels of tumor necrosis factor (TNF), angiotensin II (AngII), IL‐6, and interleukin‐8 (IL‐8), and was associated with damage to the kidneys and vascular structures [22, 27] (Figure 2). Increased tissue cellularity, reflecting inflammatory infiltration, has also been observed in the liver of HSD‐fed mice, where increased macrophage and neutrophil accumulation correlated with increased fibrosis and altered hepatic lipid content (Figure 2) [22].

Beyond acute effects, chronic high‐salt exposure induces innate immune training in hematopoietic progenitors, imprinting a durable pro‐inflammatory, hyporeparative macrophage phenotype that persists even after dietary salt withdrawal [13]. In a model of ischemic stroke, this transcriptional reprogramming resulted in metabolic changes in monocyte‐derived macrophages and was associated with poorer recovery. Together, these findings indicate that HSD can imprint long‐lasting changes on the immune cell landscape, which may critically exacerbate disease severity.

## 6 Research Gaps and Future Directions

Despite increasing research on high salt intake and noncommunicable diseases, the consequences for the gut microbiota and immune function remain poorly understood. Although HSD can profoundly alter the gut microbiota, evidence from human studies is still limited. Most available studies include small cohorts (fewer than 50 participants) and short intervention periods (2–14 weeks), which likely fail to capture long‐term effects observed in animal models, such as sustained microbiota alterations and persistent disturbances in host metabolism [23, 100]. Experimental reproducibility in animal models is further influenced by housing conditions, which strongly shape microbiota composition. The recent development of wildling mice, which carry resilient natural microbiota while retaining tractable C57BL/6 genetics, offers a promising avenue for improving both reproducibility and translational relevance in future HSD studies [41, 101].

HSD may also impair intestinal barrier integrity, thereby promoting bacterial translocation, particularly to the kidney. Whether such translocation directly activates tissue‐resident macrophage populations at the gut–liver axis, and how this intersects with the direct effects of high salt on macrophage function, remains an open and important question. Macrophages appear particularly sensitive to HSD, yet most mechanistic insights derive from in vitro systems. Particularly underexplored is how HSD affects the identity and function of tissue‐resident macrophage populations at the gut–liver axis, and whether their responses to high salt are shaped by local microbial cues, systemic metabolite changes, or direct sodium sensing.

Ultimately, dissecting the relative contributions of dysbiosis, metabolite depletion, barrier breach, and direct sodium sensing to macrophage reprogramming will require integrative experimental designs that capture these processes simultaneously. These studies will be essential for understanding how dietary salt contributes to the rising burden of noncommunicable diseases in the Global North.

## Author Contributions

A.A., M.L.D.W., M.F.V., and E.M. wrote and edited the manuscript.

## Conflicts of Interest

The authors declare no conflicts of interest.

## Data Availability

Data sharing is not applicable to this article as no new data were created or analyzed in this study.
